# Genetic and clinical spectrum of early growth response 2-related Charcot-Marie-Tooth disease in a Brazilian cohort

**DOI:** 10.1055/s-0045-1806820

**Published:** 2025-04-22

**Authors:** Eduardo Boiteux Uchôa Cavalcanti, Savana Camilla de Lima Santos, Christian Marques Couto, Galeno Vieira Rocha, Maria Cristina Del Negro Barroso Freitas, Osvaldo José Moreira do Nascimento

**Affiliations:** 1 Rede SARAH de Hospitais de Reabilitação, Ambulatório de Reabilitação Neurológica, Brasília DF, Brazil.; 2 Rede SARAH de Hospitais de Reabilitação, Labotatório de Patologia Molecular, Brasília DF, Brazil.; 3 Rede SARAH de Hospitais de Reabilitação, Ambulatório de Reabilitação Neurológica, Rio de Janeiro RJ, Brazil.; 4 Rede SARAH de Hospitais de Reabilitação, Ambulatório de Reabilitação Neurológica, Fortaleza CE, Brazil.; 5 Universidade Federal Fluminense, Hospital Universitário Antônio Pedro, Niterói RJ, Brazil.

**Keywords:** Hereditary Sensory and Motor Neuropathy, Charcot-Marie-Tooth Disease, Early Growth Response Protein 2

## Abstract

**Background** Charcot-Marie-Tooth (CMT) disease is a genetically diverse group of hereditary neuropathies. Most studies on the frequency of CMT subtypes report that the early growth response 2 ( *EGR2* ) gene accounts for less than 1% of cases. However, data regarding the epidemiology and clinical characteristics of *EGR2* -related CMT in Central and South America remain limited.

**Objective** To characterize the clinical and genetic features of *EGR2* -related CMT in a Brazilian cohort.

**Methods** We retrospectively analyzed clinical and ancillary data from four individuals with confirmed molecular diagnosis of *EGR2* -related CMT. Patients were categorized based on age of onset, motor nerve conduction velocity of the ulnar nerve, and nerve biopsy findings when available. Next-generation sequencing was utilized for genetic analysis.

**Results** Pathogenic and likely pathogenic variants were identified exclusively in the three zinc-finger domains of *EGR2* , including a novel variant, c.1234G > C (p.Glu412Gln). Patients exhibited significant variation in clinical severity and phenotypes. Dysphagia, respiratory complications, and scoliosis were prominent features.

**Conclusion** Our findings corroborate the complex and varied clinical presentations of *EGR2* -related CMT, highlighting respiratory issues and dysphagia as significant features. Comprehensive clinical assessment and early genetic diagnosis are essential for managing this condition's diverse phenotypic spectrum.

## INTRODUCTION

Charcot-Marie-Tooth disease (CMT), a group of hereditary sensory-motor neuropathies, stands as one of the most prevalent inherited neurological disorders worldwide, with an estimated prevalence ranging from 9.7 to 82.3 in 100 thousand individuals, depending on ethnic background and ascertainment method. 1 2 3 4 5 This group of disorders commonly manifests with slowly progressive distal muscle weakness and a variable degree of sensory involvement, with phenotypes that vary in severity and age of onset. 6 7 In addition, recent advances have unveiled the increasing genetic complexity underlying this group of diseases, with more than 100 genes implicated in the pathogenesis of CMT, each contributing to distinct subtypes and even overlapping with other complex neurological syndromes. 8 9 10

In 1998, Warner et al. hypothesized that the early growth response 2 ( *EGR2)* gene may act as a transcription factor affecting late myelin genes, and that human myelinopathies of the peripheral nervous system may result from disease-causing variants in this gene. 11 Since the initial description, numerous variants of the *EGR2* gene have been identified, with the majority situated within the zinc-finger (ZNF) DNA-binding domain. These variants exhibit diverse inheritance patterns, including autosomal dominant, autosomal recessive, and de novo mutations in sporadic cases. 7 12 13 14 15 16 17 18 19

Most studies on CMT subtypes show that *EGR2* accounts for less than 1% of cases. 20 21 22 23 24 Despite being a rare cause of CMT, the variable phenotypic presentation and presence of additional features, such as sensorineural hearing-loss, scoliosis, and cranial nerve involvement, can make the diagnosis challenging. 19 25

In Central and South America, information regarding the epidemiology and clinical characteristics of *EGR2* -related CMT is limited. 21 26 The present study conducted a retrospective analysis of the clinical and ancillary data from four individuals with confirmed molecular diagnosis of *EGR2* -related CMT and compared these findings with previously reported cases.

## METHODS

### Patients

We retrospectively analyzed data from four individuals with confirmed molecular diagnosis of *EGR2* -related CMT, recorded in our Neuromuscular Outpatient Registry. All individuals received their diagnosis between 2010 and 2024.

The inclusion criteria were:

- A clinical diagnosis of CMT established through clinical symptoms and neurophysiological findings;
- Confirmation of pathogenic or likely pathogenic variants in the *EGR2* gene via genetic testing; and
- Availability of detailed clinical records and ancillary test results.

The exclusion criterion was patients with a clinical diagnosis of CMT who did not have genetic confirmation of pathogenic or likely pathogenic *EGR2* variants.

The current study received approval from the Ethics Committee of Rede SARAH de Hospitais de Reabilitação in the Brazilian Federal District of Brasília, in accordance with Brazilian ethical regulations (CAAE 42388815.2.0000.0022).

### Clinical assessment

Patients were categorized based on the age of onset, motor nerve conduction velocity (MNCV) of the ulnar nerve, and, when available, nerve biopsy findings. The age of onset was determined based on the age of the first clinical symptom attributed to CMT.

The terms congenital hypomyelinating neuropathy (CHN) and Dejerine-Sottas neuropathy (DSN) have been preserved due to their frequent usage in the current medical literature. However, it is important to acknowledge that these terms represent facets of a wider phenotypic spectrum of hereditary neuropathies rather than distinct clinical entities.

The CMT Neuropathy Score version 2 (CMTNSv2) was applied to assess the severity of CMT in three subjects. 27 The score was directly administered to Subject 2, while scores for Subjects 1 and 3 were retrospectively estimated from their clinical records. This scoring system categorizes patients into mild (CMTNS ≤ 10), moderate (CMTNS 11–20), or severe (CMTNS > 20) CMT. However, it is essential to note that this scale has not yet been validated in Portuguese, necessitating the use of a free translation for this analysis.

### Neurophysiology

All patients underwent neurophysiological evaluations conducted according to standardized protocols. These assessments included sensory and motor nerve conduction velocities measurements and needle electromyography. Subjects were categorized based on the motor nerve conduction velocity (MNCV) of the ulnar nerve into 3 groups: 1) demyelinating (MNCV ≤ 35 m/s); 2) intermediate (MNCV ranging from 36–45 m/s); and 3) axonal (MNCV > 45 m/s). Reference values for nerve conduction studies are provided in **Supplementary Material Table S1** (available at https://www.arquivosdeneuropsiquiatria.org/wp-content/uploads/2025/01/Supplementary-Material-Table-S1.xlsx ).

### Next-generation sequencing and analyses

Genomic DNA was extracted from peripheral blood samples using a standard protocol. For initial screening of DNA samples from all patients diagnosed with demyelinating CMT, multiplex ligation-dependent probe amplification (MLPA) was used to detect duplications or deletions in the *PMP22* gene.

In suspected cases of demyelinating CMT without male-to-male inheritance or sporadic cases lacking *PMP22* duplication ( *PMP22* dup), Sanger sequencing was performed to analyze the *GJB1* gene. Intermediate and axonal CMT cases, as well as those negative for *PMP22* dup and pathogenic or likely pathogenic *GJB1* variants, were further assessed using a genetic panel covering 72 genes ( **Supplementary Material Table S2** –available at https://www.arquivosdeneuropsiquiatria.org/wp-content/uploads/2025/01/Supplementary-Material-Table-S2.xlsx ).

Next-generation sequencing (NGS) was conducted using the SureSelect QXT and QXT HS2 Library Prep Kit (Agilent Technologies, Santa Clara, CA, USA) on an Illumina MiSeq sequencer (Illumina, Inc., San Diego, CA, USA). The sequencing data were processed with the Illumina Pipeline (Illumina), using the hg19 genome as a reference. Bioinformatic analysis included base calling, read alignment, variant identification, and variant annotation through the Varstation v.3.0 software. 28

To further investigate the inheritance patterns of identified variants, we performed Sanger sequencing on the parents of subject 2 and the grandparents of subject 1 to detect the c.1142G > A (p.Arg381His) variant. However, due to logistical challenges and difficulties in coordinating appointments, testing for the c.1066G > A (p.Glu356Lys) variant in subject 3 and the c.1234G > C (p.Glu412Gln) variant in subject 4 was not feasible in their parents, as the patients and their families live at considerable distances from the hospital facilities.

All identified variants were classified according to the American College of Medical Genetics (ACMG) standards and guidelines. Cases with pathogenic and likely pathogenic variants were considered genetically confirmed.

## RESULTS

### Clinical picture and ancillary tests

**Figure 1:**
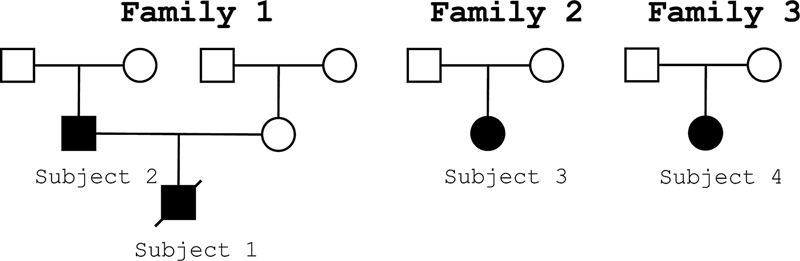
Heredogram illustrating inheritance patterns and family histories in early growth response 2 ( *EGR2* )-related neuropathy.

Four patients with *EGR2* -related hereditary neuropathy were thoroughly evaluated. A detailed pedigree chart was created to illustrate the inheritance patterns and document the family histories ( Figure 1 ). Clinical features, nerve conduction studies, and genetic analyses of the subjects are summarized in Table 1 .

**Table 1:** Clinical characteristics of patients with *EGR2* -related Charcot-Marie-Tooth disease

Characteristics	Subject 1	Subject 2	Subject 3	Subject 4
Sex	Male	Male	Female	Female
Age at onset (years)	2	30	0	11
Delivery route	Normal	Normal	C-section	Normal
Age at first evaluation (years)	6	40	3	21
Family history	Autosomal dominant	Autosomal dominant	Sporadic	Sporadic
Scoliosis	+	−	+	−
Foot deformity (pes cavus)	+	+	+	+
Brainstem auditory evoked potential	Normal	Normal	Abnormal	Normal
Visual evoked potential	Normal	Normal	N/A	N/A
Pulmonary function	Individual dependent on mechanical ventilation/tracheostomized	Mild restrictive ventilatory defect (FVC 80%/FEV1 67%)	N/A	N/A
Dysphagia	+	−	−	−
Ulnar MNCV (m/s)	10.1	28.3	6.9	43
Ulnar CMAP (mV)	0.3	2.2	0.5	2.7
NGS-CMT Panel	c.1142G > A (p.Arg381His)	c.1142G > A (p.Arg381His)	c.1066G > A (p.Glu356Lys)	c.1234G > C (p.Glu412Gln)
CMTNS - version 2	33 (severe)	14 (Moderate)	32 (severe)	N/A
Phenotype	Familial CMT1D	Familial CMT1D	Sporadic CHN case	Sporadic CMT1D case

Abbreviations: CMAP, compound motor action potential; CMTNS, Charcot-Marie-Tooth Neuropathy Score; *EGR2* , early growth response 2; MNCV, motor nerve conduction velocity; NGS, next-generation sequencing.

**Figure 2:**
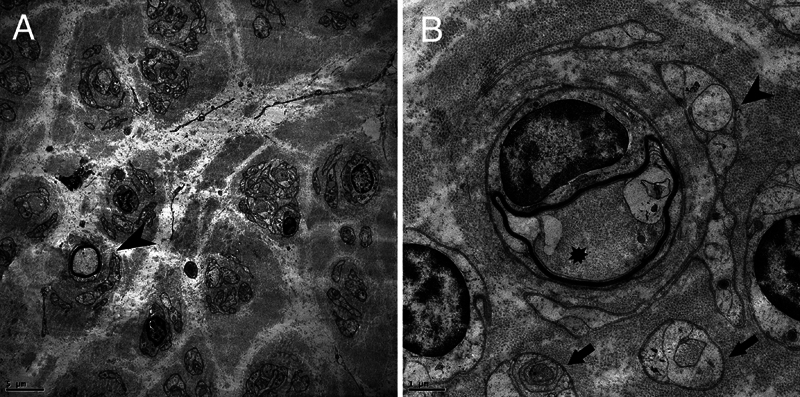
Electron microscopy of the sural nerve biopsy. ( **A** ) Region demonstrating loss of myelinated fibers (arrowhead indicates a thinly myelinated fiber). ( **B** ) Axon (asterisk) surrounded by concentric layers of Schwann cell laminae (arrowhead) enveloping bundles of collagen fibers (empty collagen pockets indicated by arrow).

Subject 1 was first evaluated in our service at age 6 years, due to a history of frequent trips and falls after starting to walk independently at 14 months. Over time, he developed dyspnea, hypophonia, scoliosis, and finger contractures, eventually requiring walking aids due to foot drop. Vocal cord paralysis was considered, but an otorhinolaryngologist did not evaluate the patient. At the age of 13, he suffered a cardiac arrest during pneumonia treatment, leading to permanent ventilatory assistance, loss of independent walking, and placement of a gastrostomy. He died at the age of 18 due to complications from surgery to repair a chronic pneumothorax. Despite being classified as CMT1D, this patient's disease course was marked by severe respiratory compromise, requiring permanent invasive ventilatory support after a cardiac arrest. Postmortem nerve biopsy revealed features of CMT, including a marked reduction in myelin fiber density, Schwann cell abnormalities, severe fiber loss, collagen pockets, Büngner bands, rare regenerative groups, and occasional macrophages and mast cells ( Figure 2 ).

Subject 2, 46-year-old man and the father of subject 1, presented with a decade-long history of distal leg numbness, ankle twisting, and foot deformity. Physical examination revealed pes cavus and mild weakness of the lower limbs, along with absent ankle reflexes and reduced pinprick and vibration sensitivity. Ancillary tests indicated vitamin B12 deficiency and mild restrictive lung disease.

Subject 3, a 15-year-old female, exhibited hypotonia at birth, delayed motor milestones, and recurrent pneumonia episodes. Speech therapy evaluation indicated frequent choking, cyanosis, and respiratory distress during meals, primarily in the later stages, due to muscular fatigue without coordination disorders. Subsequently, she developed scoliosis and progressive dysphagia requiring gastrostomy. The patient presented respiratory symptoms, including episodes of orthopnea and dyspnea in some daily tasks. Although a diagnosis of obstructive sleep apnea was reported from an external service, the polysomnography was unavailable for analysis. The chest computed tomography (CT) revealed significant scoliosis of the thoracic spine with leftward convexity, accompanied by rib cage deformity and reduced left lung volume. Linear atelectasis was observed in the posterior lung bases. The esophagram showed multiple gastroesophageal reflux episodes extending to the thoracic esophagus's upper third, with rapid clearance. Additional tests indicated partial bilateral involvement of auditory evoked potentials. There was no family history of neuromuscular disorders, and her parents were non-consanguineous and asymptomatic. The patient was classified as CHN.

Subject 4, a 21-year-old female, presented at age 12 with a history of stumbling, leg tingling, and pes cavus, accompanied by urinary urgency. At her last appointment, the patient was using ankle-foot orthoses as a mobility aid. There was no family history of neuromuscular disorders, and her parents were non-consanguineous and asymptomatic.

### CMTNSv2

The CMTNSv2 was evaluated in three patients, with findings detailed in Table 1 and **Supplementary Material Table S3** (available at https://www.arquivosdeneuropsiquiatria.org/wp-content/uploads/2025/01/Supplementary-Material-Table-S3.xlsx ). Subjects 1 and 3 were classified as having severe CMT (CMTNSv2 > 20), while subject 2 was classified as having moderate CMT (CMTNSv2: 11–20).

### Neurophysiology

Nerve conduction studies are consistent with CMT1 in 3 subjects, with ulnar nerve MNCV ≤ 35 m/s. Furthermore, very slow MNCV (≤ 5 m/s) were observed in individuals 1 and 3. Subject 4 presented MNCV in the intermediate range (36–45 m/s) ( Table 2 ).

**Table 2:** Nerve conduction study findings in subjects with *EGR2* -related Charcot-Marie-Tooth disease

Subject	Age (years)	Motor nerves	CMAP (mV)	Distal latency (ms)	MNCV (m/s)	Sensory nerves	SNAP (µV)
Subject 1	7	Median	N/A	N/A	N/A	Radial	NR
		Ulnar	0.3	N/A	10.1	Ulnar	N/A
		Peroneal	N/A	N/A	N/A	Sural	NR
		Tibial	N/A	N/A	N/A		
Subject 2	41	Median	0.73	17.1	29.1	Radial	NR
		Ulnar	1.3	12.3	28.3	Ulnar	NR
		Peroneal	1.77	5.44	N/A	Sural	NR
		Tibial	N/A	N/A	N/A		
Subject 3	3	Median	0.2	33.6	6.8	Radial	NR
		Ulnar	0.5	32.2	6.9	Ulnar	NR
		Peroneal	NR	NR	NR	Sural	NR
		Tibial	NR	NR	NR		
Subject 4	22	Median	4.4	12.24	45	Radial	N/A
		Ulnar	2.7	10.63	43	Ulnar	18.1
		Peroneal	NR	NR	NR	Sural	NR
		Tibial	NR	NR	NR		

Abbreviations: CMAP, compound muscle action potential; *EGR2* , early growth response 2; MNCV, motor nerve conduction velocity; SNAP, sensory nerve action potential; NR, non-recordable; N/A, not available.

### Next-generation sequencing and analyses

**Figure 3:**
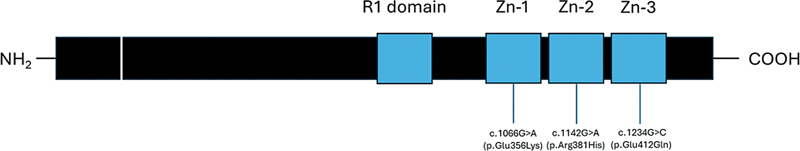
Schematic representation of the *EGR2* gene and the identified variants.

Panel-based next-generation sequencing (NGS) identified pathogenic or likely pathogenic mutations in all four subjects, revealing three different variants ( Table 3 and Figure 3 ).

**Table 3:** Genetic variants identified in subjects with *EGR2* -related Charcot-Marie-Tooth disease

Subject	Phenotype	SeqRef	Variant	State	ACMG criteria	Classification	Reference
1	CMT1D	NM_000399.5	c.1142G > A (p.Arg381His)	Heterozygous	PM1 PM2 PM5 PP5 PS2 PS3	Pathogenic	30
2	CMT1D	NM_000399.5	c.1142G > A (p.Arg381His)	Heterozygous	PM1 PM2 PM5 PP5 PS2 PS3	Pathogenic	30
3	CHN	NM_000399.5	c.1066G > A (p.Glu356Lys)	Heterozygous	PM1 PM2 PM5 PP5 PS2	Likely pathogenic	33
4	CMT1D	NM_000399.5	c.1234G > C (p.Glu412Gln)	Heterozygous	PM1 PM2 PM5	Likely pathogenic	This work

Abbreviations: ACMG, American College of Medical Genetics; *EGR2* , early growth response 2; PM, pathogenic moderate; PP, pathogenic supporting; PS, pathogenic strong.

The first variant, c.1142G > A (p.Arg381His), was identified in a heterozygous state in subjects 1 and 2, who were part of a previous study on the frequency of genetic subtypes of CMT published by our group. 21 Sanger sequencing did not detect this variant in the parents of subject 2, suggesting a *de novo* mutation. It is not reported in gnomAD exomes and genomes. Most algorithms developed to predict the effect of missense changes on protein structure and function suggest that this variant is likely to be disruptive. Assessment of experimental evidence suggests that this variant results in abnormal protein function. 29 This variant has been previously described in medical literature and is associated with different phenotypes of hereditary neuropathies. 21 25 30 31 It is classified as pathogenic.

The second variant, c.1066G > A (p.Glu356Lys), was found in a heterozygous state in subject 3. Although this variant is not reported in gnomAD exomes and genomes, it is classified as likely pathogenic according to the ACMG standards and guidelines. It was previously identified in a Chinese cohort of CMT patients without a detailed phenotype description. 32 It has also been reported in patients with chronic lymphocytic leukemia (CLL) and associated with a shorter time to treatment and poor overall survival, but without clinical diagnosis of CMT. 33 Predictive algorithms have conflicting results on this variant's potential for disruption, and functional studies have yet to confirm these predictions.

The third and novel variant, c.1234G > C (p.Glu412Gln), was detected in a heterozygous state in subject 4. This variant is also unreported in gnomAD exomes and genomes and is classified as likely pathogenic according to the ACMG standards and guidelines. Predictive algorithms have conflicting results regarding this variant's potential to be disruptive. Functional studies have not yet confirmed these predictions. This specific variant has not been described in the medical literature, but similar amino acid changes at the same codon (c.1235A > G (p.Glu412Gly) and c.1234G > A (p.Glu412Lys)) have been reported. 32 34 35

## DISCUSSION

Our study provides valuable insights into the clinical and genetic spectrum of *EGR2* -related CMT disease in a Brazilian cohort, addressing a significant gap in epidemiological data for this condition in Central and South America. Notably, only two studies have previously reported on the frequency of genetic subtypes of CMT in Brazilian clinical populations, underscoring the limited data on *EGR2* -related CMT in the region. 21 26 We identified a novel variant, c.1234G > C (p.Glu412Gln), within the ZNF domain of *EGR2* , expanding the known mutational spectrum of this gene. This discovery, along with the significant clinical features observed, such as respiratory complications and dysphagia, underscores the complex nature of EGR2-related CMT.

Most patients with *EGR2* -related CMT have mutations in the ZNF DNA-binding domain. 13 14 15 16 17 18 19 The *EGR2* gene encodes a C _2_ H _2_ -type zinc-finger transcription factor that regulates the expression of genes involved in myelin formation and maintenance, including myelin-associated glycoprotein ( *MAG* ), myelin basic protein ( *MBP* ), myelin protein zero ( *MPZ* ), gap junction beta 1 ( *GJB1* ), peripheral myelin protein ( *PMP22* ), and periaxin ( *PRX* ). 12 13 The variation in clinical severity seen with disease-causing variants in the ZNF domain appears to correlate with the level of residual DNA binding. 12

To date, only two variants outside the three-ZNF DNA-binding domain have been identified, both located in the R1 domain of *EGR2* and following an autosomal recessive inheritance pattern. 11 14 In those cases, the proposed mechanism is related to the inhibition of the interaction of *EGR2* with NAB corepressors, which are necessary for the myelination process. 11 14

In our study, all identified pathogenic and likely pathogenic variants were within the three ZNF regions ( Table 3 ). The discovery of the novel likely pathogenic variant c.1234G > C (p.Glu412Gln) in the ZNF3 domain of *EGR2* expands our understanding of the mutational spectrum underlying *EGR2* -related CMT. However, additional functional studies are required to elucidate the precise molecular consequences of this variant.

Phenotypic variability is observed among patients with *EGR2* -related CMT, highlighting the heterogeneous nature of this condition. 7 14 15 16 17 18 Our data corroborate previous findings, with three individuals classified as CMT1D and one as CHN. This variability underscores the importance of considering both clinical and genetic factors in disease classification and prognosis, emphasizing the need for individualized therapeutic approaches.

Subjects 1 and 2 exhibited restrictive patterns on pulmonary function tests, indicating respiratory muscle weakness—a recognized complication in CMT. 21 25 36 Notably, subject 1's disease course was unusually severe for CMT1D, progressing to respiratory failure and requiring permanent ventilatory support following a cardiac arrest during an episode of pneumonia. This atypical severity may be related to the *EGR2* variant c.1142G > A (p.Arg381His), which has been associated with severe CMT phenotypes, including respiratory complications. 25 In contrast, subject 2, his father, who carries the same mutation, presented with a milder phenotype, limited to distal leg weakness and mild restrictive pulmonary changes. This intrafamilial variability suggests the influence of additional genetic, epigenetic, or environmental modifiers in *EGR2* -related neuropathies. Similarly, the respiratory symptoms and recurrent pneumonia in subject 3 highlight the importance of proactive respiratory monitoring and management in these patients, given the broad phenotypic spectrum even within families.

Dysphagia, another significant clinical feature observed in subjects 1 and 3, is likely due to oropharyngeal muscle weakness, a recognized complication of certain CMT subtypes. 37 Bulbar weakness increases the risk of aspiration and nutritional deficits, contributing to the overall disease burden. Multidisciplinary care is essential to enhance quality of life, prevent life-threatening respiratory complications, and minimize the impact of dysphagia and bulbar involvement in patients with *EGR2* -related CMT.

Peripheral nerve biopsies in *EGR2* -related neuropathies commonly show a combination of demyelinating and axonal degeneration. 18 34 35 Most important findings include thinning of myelin sheaths, frequent onion bulb formations, and axonal loss with regenerative clusters. Schwann cell morphological abnormalities are also prominent, reflecting *EGR2* 's critical role in Schwann cell differentiation. Additionally, some patients may present with a predominantly axonal phenotype, characterized by significant axonal degeneration with less pronounced demyelination, highlighting the variability in pathological manifestations of *EGR* 2-related neuropathies. 34 35

Our study has limitations, including a small sample size and retrospective design that must be considered. Larger, prospective studies are essential to validate our findings and further explore genotype-phenotype correlations. Furthermore, functional characterization of the novel variant identified will be crucial to understand its pathogenicity and molecular mechanisms.

Future research should focus on conducting functional studies to determine the impact of the novel and other identified variants on *EGR2* function. Specifically, transactivation assays could evaluate the transcriptional activity of these variants, while electrophoretic mobility shift assays (EMSA) would assess their DNA-binding capabilities. 12 18 38 Developing animal models to investigate the in vivo effects of *EGR2* mutations could provide insights into their role in myelination processes. Furthermore, research on effective management strategies for respiratory and swallowing complications in *EGR2* -related CMT patients is also necessary. Collaborative efforts are needed to gather more extensive data on *EGR* 2-related CMT in diverse populations, particularly in underrepresented regions like Central and South America.

In conclusion, our findings highlight the heterogeneity in disease severity among individuals with EGR2-related CMT, emphasizing the need for a multidisciplinary approach to patient care. Early genetic diagnosis and comprehensive clinical assessment are critical for managing this condition's diverse phenotypic spectrum. By advancing our understanding of *EGR2* -related CMT, we can improve outcomes for affected patients and their families.
